# P-974. Barriers and Facilitators to Infectious Disease Consultation for Patients with *Staphylococcus aureus* Bacteremia in Rural Hospitals: a Qualitative Pilot Study

**DOI:** 10.1093/ofid/ofaf695.1173

**Published:** 2026-01-11

**Authors:** Laurel Legenza, Linda McKinley, Marin Schweizer, Nasia Safdar, Casper G Bendixsen, Julie Keating

**Affiliations:** University of Wisconsin-Madison School of Pharmacy, Madison, Wisconsin; Wm. S. Middleton Memorial VA Hospital, Madison, Wisconsin; William S. Middleton VA Hospital, madison, Wisconsin; University of Wisconsin School of Medicine and Public Health, Madison, WI; Marshfield Clinic Research Institute, Marshfield, Wisconsin; Madison VA Hospital, Madison, Wisconsin

## Abstract

**Background:**

*Staphylococcus aureus* bacteremia (SAB) is associated with multiorgan complications and a high mortality. Infectious disease (ID) consultation improves outcomes for patients with SAB and is recommended by Infectious Diseases Society of America guidelines however, many rural hospitals lack on-site ID specialists.

**Methods:**

We conducted a qualitative pilot project with Wisconsin hospitals serving rural patients to understand current processes and needs for obtaining ID consultation for patients with SAB. Interviews were conducted with healthcare personnel with SAB care experience. We also collected feedback on an electronic alert aimed at promoting use of ID consultation for SAB management. Data was analyzed with a rapid qualitative inquiry approach.

**Results:**

We interviewed six participants (3 pharmacists, 2 infection preventionists, and 1 laboratory manager) across six hospitals. While no hospitals had full-time in-house ID consultants, all sites had local pharmacist support including local or health system antimicrobial stewardship (AMS) teams that assisted in SAB management. Access to and processes for seeking ID consultation varied between sites, including consult formality and local presence of the provider. Barriers to SAB care included variable provider experience and urgency with SAB management and communication limitations. Some providers described knowledge gaps around SAB complexity of sequalae and need for follow-up. Participants liked the alert design but noted challenges with implementing alerts, including alert fatigue and bureaucracy in electronic health record approvals particularly within shared networks.

**Conclusion:**

Local variability in processes limits the utility of electronic alerts for promoting ID consultation. In-person ID consultation is rare in rural hospitals. A telehealth or automated alert that provides guidance on urgency, first-line therapy, and communication may promote guideline-concordant SAB care within the unique workflows of small rural hospitals. Variability in processes and provider urgency is a gap for first-line treatment and critical post-SAB diagnosis care. Given the presence and existing roles of pharmacists in SAB management, SAB decision support and management could include the local pharmacist and AMS team.

**Disclosures:**

Linda McKinley, RN, PhD, MPH, CIC, FAPIC, Molnlycke: Advisor/Consultant

